# A New Species of Skin-Feeding Caecilian and the First Report of Reproductive Mode in *Microcaecilia* (Amphibia: Gymnophiona: Siphonopidae)

**DOI:** 10.1371/journal.pone.0057756

**Published:** 2013-03-06

**Authors:** Mark Wilkinson, Emma Sherratt, Fausto Starace, David J. Gower

**Affiliations:** 1 Department of Zoology, The Natural History Museum, London, United Kingdom; 2 Department of Organismic and Evolutionary Biology and Museum of Comparative Zoology, Harvard University, Cambridge, Massachusetts United States of America; 3 BP 127, 97393 Saint Laurent du Maroni Cedex, French Guiana; University of Sao Paulo, Brazil

## Abstract

A new species of siphonopid caecilian, *Microcaecilia dermatophaga* **sp. nov.**, is described based on nine specimens from French Guiana. The new species is the first new caecilian to be described from French Guiana for more than 150 years. It differs from all other *Microcaecilia* in having fewer secondary annular grooves and/or in lacking a transverse groove on the dorsum of the first collar. Observations of oviparity and of extended parental care in *M. dermatophaga* are the first reproductive mode data for any species of the genus. *Microcaecilia dermatophaga* is the third species, and represents the third genus, for which there has been direct observation of young animals feeding on the skin of their attending mother. The species is named for this maternal dermatophagy, which is hypothesised to be characteristic of the Siphonopidae.

## Introduction

Kupfer *et al.* [1] discovered a novel form of extended parental care in the oviparous African herpelid caecilian *Boulengerula taitanus* in which altricial hatchlings feed upon the modified and lipid-rich outer layer of the skin of their attending mothers using a specialised deciduous juvenile dentition. Subsequently, Wilkinson *et al.* [2] reported the putatively homologous behaviour and associated morphological and physiological features of maternal dermatophagy in a second species of caecilian, the Neotropical siphonopid *Siphonops annulatus*. Because these two species of skin-feeding caecilians are not particularly closely related and represent lineages that have been separated for more than 100 million years, Wilkinson *et al.* [2] suggested that skin feeding was a relatively ancient trait and predicted that it would prove to be more widespread among caecilians.

The Neotropical siphonopid genus *Microcaecilia* Taylor, 1968 includes eight previously described nominal species of relatively small caecilians with heavily ossified, stegokrotaphic skulls, and small eyes that are covered with bone [3] which suggest they are dedicated burrowers. Very little is known of their biology and there are no previous reports of the reproductive biology of any *Microcaecilia*. Here we describe a new species of *Microcaecilia* from French Guiana. Observations of reproduction in captivity reveal that this is a third caecilian species known from direct observation to practice maternal dermatophagy. The species is identified as a member of the Siphonopidae on the basis of being an oviparous caecilian with imperforate stapes and no inner mandibular teeth, and as a *Microcaecilia* on the basis of having eyes under bone, tentacular apertures closer to the eyes than the nares, and no diastemata between the vomerine and palatine teeth [4].

## Materials and Methods

Animals were obtained by digging with bladed hoes in forest soils, especially between buttress roots of trees and under rotting wood and exclusively during daylight hours. All necessary permits for the fieldwork and export of specimens were obtained from the authority DIREN Guyane. This field study did not involve endangered or protected species. No ethical approval was required for this study because no experimentation or manipulations were carried out and there is no relevant legislation. Specimens were killed by anaesthesia (MS222), fixed in 5% unbuffered formalin for at least 2 days, washed in water and stored in 70% industrial methylated spirits. The following procedures were done on dead, alcohol-preserved specimens.

Total lengths and circumferences were measured to the nearest millimetre (mm) with a ruler, the latter by wrapping a piece of string around the body. Other measurements were made to the nearest 0.1 mm with dial callipers. Observations and counts of teeth were facilitated by using a directed steam of compressed air to temporarily dry and shrink the gingivae, a method we learnt from Ronald A. Nussbaum (University of Michigan) and which we denote the Nussbaum technique. Dermal scale pockets were opened by bending the specimen perpendicular to the long axis of the body so as to put the skin covering the opening of a pocket under tension and using a pin to perforate this skin. Subdermal scales were searched for using a scalpel to cut and reflect sections of dermis between annular grooves, and a pin to probe the underlying connective tissue. Sex was determined by dissection and direct examination of gonads. Specimens were classified as immature when gonads could not be detected.

Following Kamei *et al.* [5] and Wilkinson and Kok [6] we use the following abbreviations for anatomical features and ratios of measurements: AG = annular groove; AM = anteromedial limit of the mouth on the upper jaw; CM = corner of the mouth; C1 = first collar; C2 = second collar; NG1 = first nuchal groove (between head and collars); NG2 = second nuchal groove (between first and second collars); NG3 = third nuchal groove (between collars and anteriormost annulus); PA = primary annulus; PAG = primary annular groove; SAG = secondary annular groove; ST = snout tip; TA = tentacular aperture; TG = transverse groove (on dorsal surface of collar); L/H = total length divided by head length (the latter = distance between ST and NG1 directly behind CM); L/W = total length divided by midbody width. Distances between structures or points of reference are indicated with a dash (e.g. AM-ST = the distance between the AM and ST, which is sometimes referred to as the projection of the snout). Abbreviations of institutions are as follows: BMNH - The Natural History Museum, London; MNHNP - Muséum national d’Histoire naturelle, Paris; MPEG - Museu Paraense Emílio Goeldi, Belem. Observations were made with the assistance of a dissecting microscope. Vertebrae were counted from X-radiographs.

Live animals were maintained in captivity between 22 and 26°C on a 12 hour reverse light cycle, in moist sterilised topsoil, with some pieces of wood providing shelters on the surface, and fed *ad libitum* with earthworms and occasionally crickets. An artificial “nest” was constructed as a compact depression in soil covered entirely with a transparent piece of plastic with a piece of wood covering the plastic. Parent-offspring interactions were observed after removal of the wood, either during “daylight” or with a red light at “night”. Masses and total lengths of live animals were recorded intermittently.

### *Microcaecilia dermatophaga* sp. nov. urn:lsid:zoobank.org:act:D98DF0FB-7BD9-4955-8E76-1E90F35BB9F2

**Figure 1:**
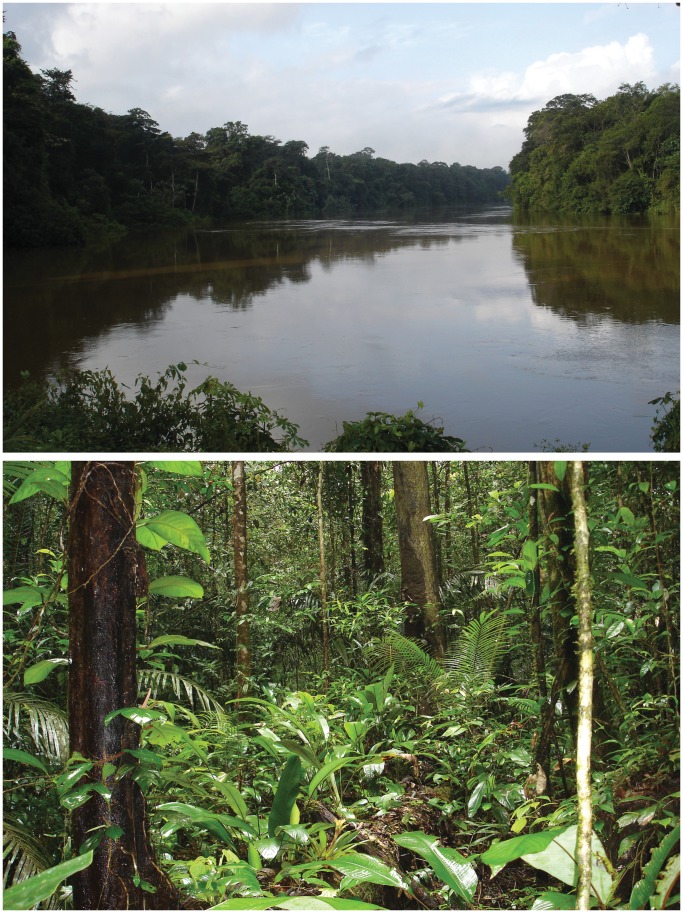
Type locality of *Microcaecilia dermatophaga* sp. nov. Forest (below) close to the Mana River (top) at Angoulême, French Guiana.

**Figure 2:**
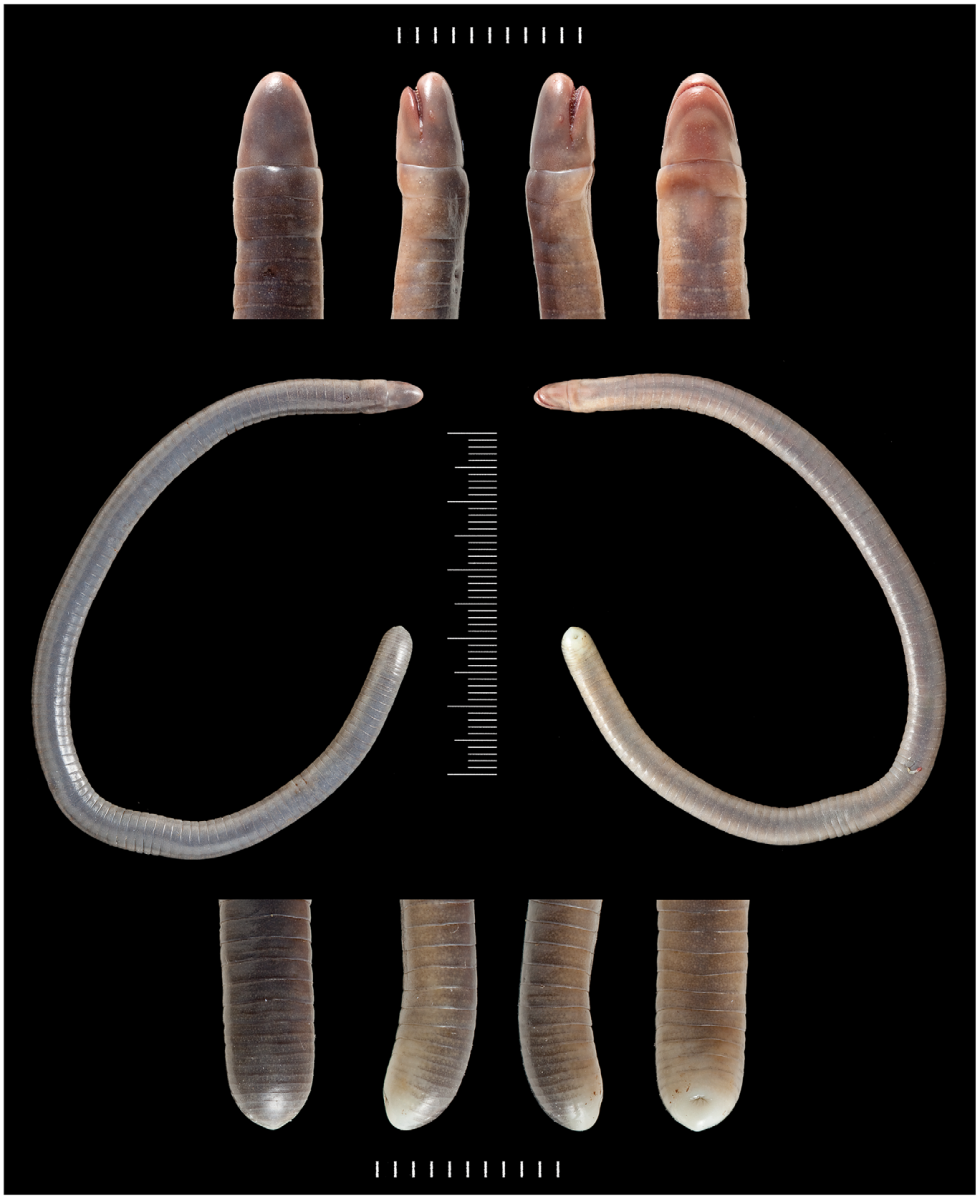
BMNH 2008.715, holotype of *Microcaecilia dermatophaga* sp. nov. Scale bars in mm.

**Figure 3:**
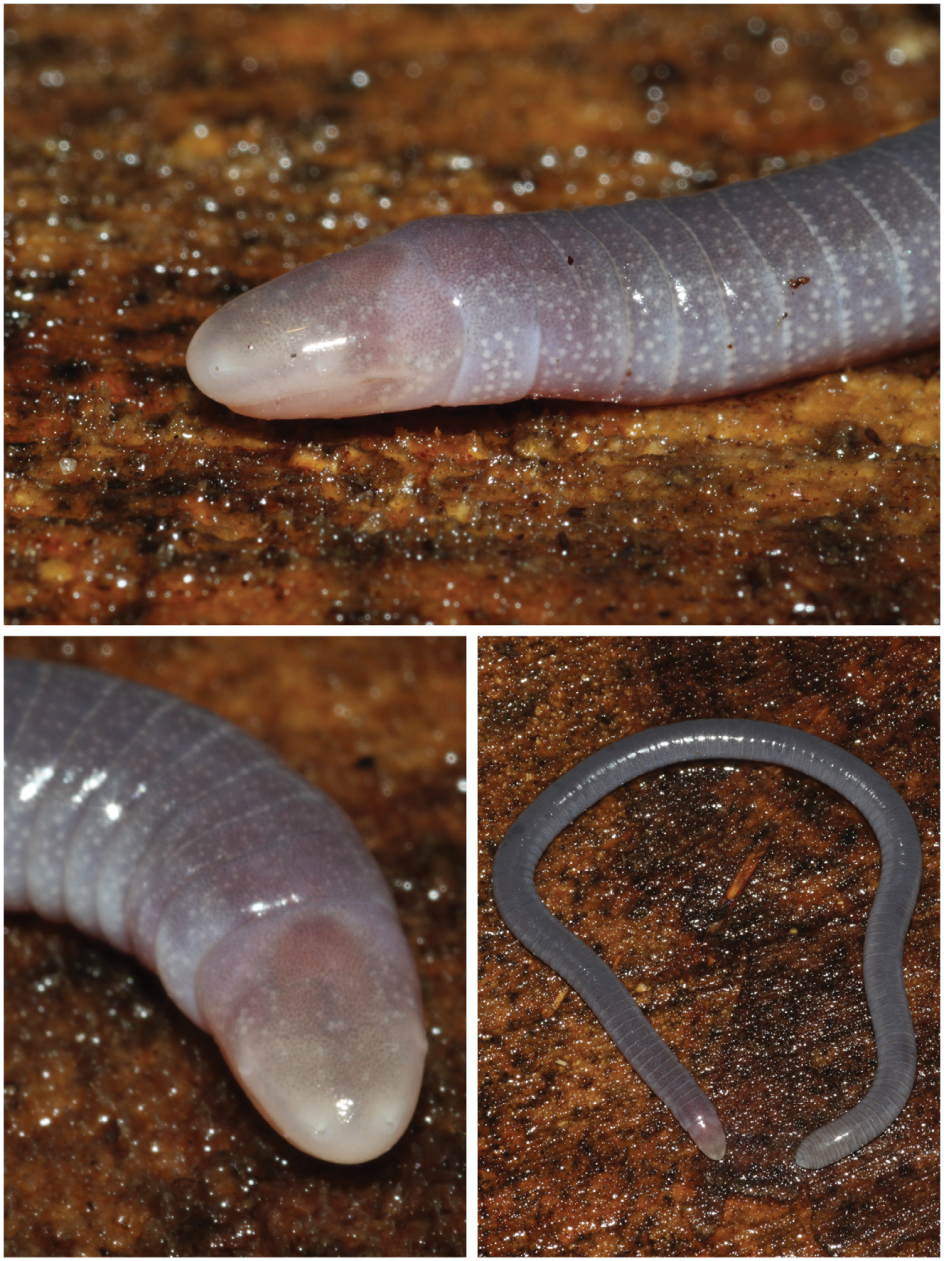
Holotype of *Microcaecilia dermatophaga* sp. nov. (BMNH 2008.715) in life.

**Figure 4:**
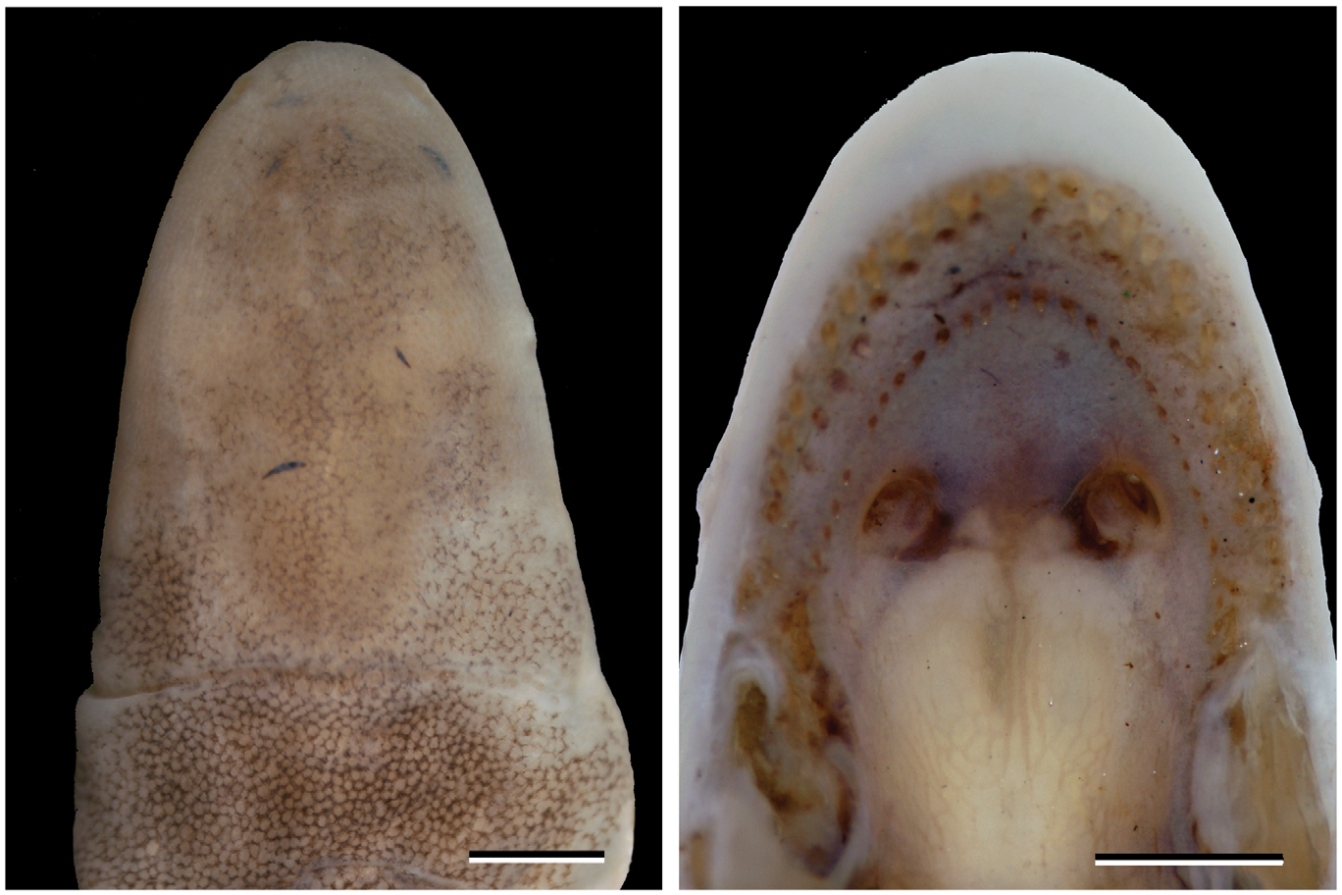
*Microcaecilia dermatophaga* sp. nov. head in dorsal and palatal views. Dorsal view of head of MNHNP 2010.0190 (left) showing six separate arthropodan exoskeletal remains (mouthparts of termites?) embedded in the skin. Palatal view of BMNH 2008.721 (right) showing disposition of tooth rows and choanae in the upper jaw. Scale bars = 1 mm.

**Figure 5:**
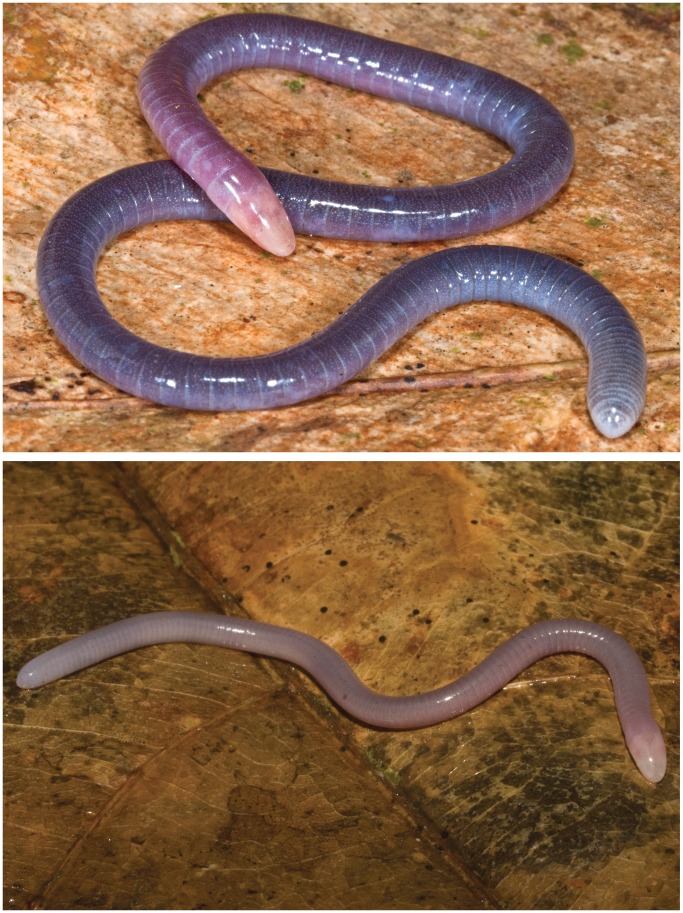
Adult and juvenile *Microcaecilia dermatophaga* sp. nov. in life. BMNH 2008.721 (top) and one of the three immature paratypes of *Microcaecilia dermatophaga* sp. nov. (bottom).

**Figure 6:**
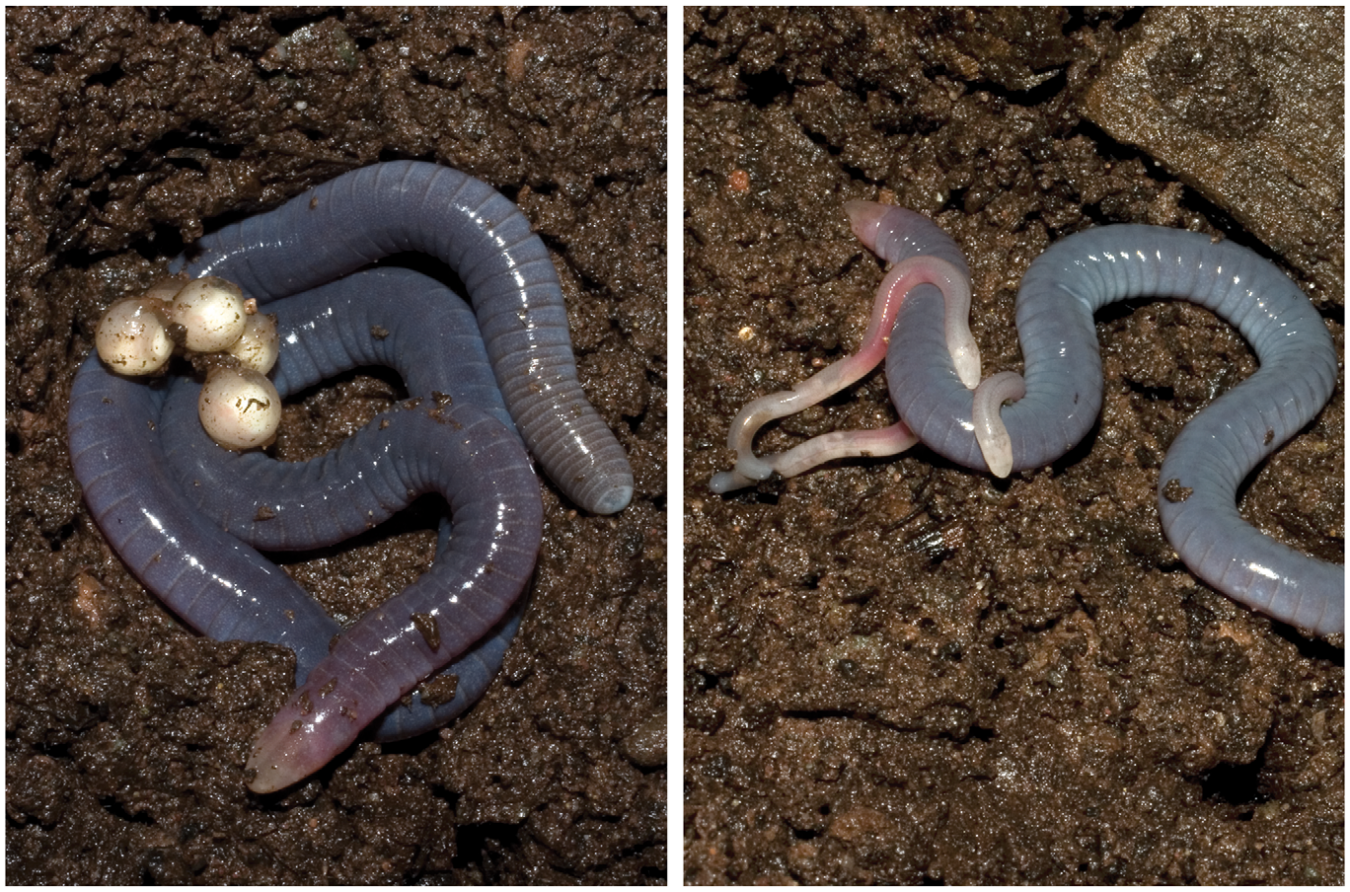
Reproductive mode of *Microcaecilia dermatophaga* sp. nov. Presumed mother with a connected string of five eggs (left) and with two hatchlings during the period of extended post-hatching parental care and maternal dermatophagy (right).

(Figs. 1, 2, 3, 4, 5, 6; Table 1).

**Table 1:** Morphometric and meristic data for the type series of *Microcaecilia dermatophaga* sp. nov.

	BMNH								MNHNP
	2008.715*	2008.716	2008.717	2008.718	2008.719	2007.720	2008.721	2008.722	2010.0190
Sex	♂	♂	♀	–	–	–	♂	♂	♂
Total length	164 (181)	152 (175)	156 (183)	66 (74)	74 (83)	75 (85)	154	148	151 (175)
PAGs ( = PAs)	108	109	109	113	109	111	112	107	111
SAGs	9	7	7	6	8	7	7	8	6
SAGs complete ventrally	0	0	0	0	0	0	0	3	0
Vertebrae	115	115	117	119	116	116	118	114	117
ST - CM	4.6	–	3.7	2.6	2.5	2.7	3.9	4.2	3.9
ST - N1 (at level of CM)	5.8	–	4.8	3.5	3.4	3.6	5.4	5.5	5.2
Head width at CM	4.2	3.8	3.4	2.4	2.4	2.4	3.5	3.4	3.4
Head with at occiput	4.6	3.9	3.8	2.5	2.5	2.6	3.7	3.6	3.5
Width at mid-body	5.0	4.1	4.1	2.5	2.6	2.7	4.1	3.8	4.4
Length of body behind vent	1.8	1.4	1.2	0.6	1.0	0.7	1.4	1.0	c. 1.2
ST – AM	1.1	–	1	0.8	0.8	1	1	1	0.9
Distance between nares	1.5	–	1.4	1	1	1	1.4	1.3	1.3
Naris – CM	3.8	–	3	1.9	2	2.2	3.4	3.6	3.3
Naris – Lip	1.0	–	0.9	0.8	0.7	0.8	0.9	0.9	1
Naris – TA	2.1	–	1.7	1.3	1.2	1.3	2	2.3	2.0
TA – TA	3.7	–	3.0	2	2	2.2	3.3	3.3	3.0
TA – CM	1.5	1.3	1.3	0.8	0.7	0.7	1.3	1.3	1.3
TA – Lip	0.5	0.4	0.4	0.3	0.3	0.3	0.4	0.4	0.4
Length of C1	1.8	1.7	1.6	1.2	1.1	1.1	1.8	1.6	1.8
Length of C2	2.1	2.1	1.8	1.3	1.3	1.3	1.8	1.8	1.9
Premaxillary-maxillary teeth	28	–	28	24	23	24	29	31	29
Vomeropalatine teeth	31	–	28*	16	19	19	32	28	27
Dentary teeth	22	20	19	18	20	20	22	22	19

* = holotype. All measures are in mm. Abbreviations given in text. Specimens without sex data are immature. Numbers in parentheses are lengths of living specimens.

#### Holotype

BMNH 2008.715, a mature male collected by Emma Sherratt, David J. Gower and Mark Wilkinson, dug from soil in forest close to Angoulême (5° 24′ 28″ N, 53° 39′ 12″ W, 55 m asl), just over 40 km East of St Laurent, French Guiana, either 10^th^ - 11^th^ May 2008 or 24^th^ - 25^th^ April 2010 and maintained alive in captivity until July 2012. The type locality (Fig. 1) is a forested area in the catchment of small streams feeding the Mana river.

#### Paratypes

BMNH 2008.716 - 720 and MNHNP 2010.0190, same data as holotype, collected 10^th^ - 11^th^ May 2008; BMNH 2008.721, same data as holotype, collected 24^th^ - 25^th^ April 2010; BMNH 2008.722, collected by Fausto Starace, 23 May 2009, found under a fallen tree in primary forest, close to the eastern outskirts of Saint Laurent du Maroni, about 40 km west of the type locality (05° 29′ 12.1″ N, 53° 59′ 34.4″ W, ca. 45 m a.s.l.).

#### Diagnosis

A *Microcaecilia* that differs from *M. taylori* in lacking a TG on C1 and from all other *Microcaecilia* in having fewer (<20) PAs that are divided by SAGs.

#### Description of the holotype

Good condition, a c. 30 mm midventral incision c. 95 mm behind snout tip, and an opened scale pocket posteriorly (Fig. 2). Some morphometric and meristic data are in Table 1.

Body somewhat dorsoventrally flattened throughout (width and depth at midbody 5.0×4.1 mm), relatively uniform, narrowing substantially only posteriorly, from just in front of the vent; L/W c. 34. In dorsal view, head much more U- than V-shaped, sides of head curve and converge gently from to back of head to level of TAs, more strongly to level of nares, ST moderately bluntly rounded. In lateral view, top of head somewhat convex; margins of upper jaw (lip) concave, somewhat downturned anterior to halfway between nares and TAs; ridge bearing vomeropalatine teeth just visible close to CM; lower jaw robust, two-thirds to four-fifths height of upper jaw at levels of CM and TA. In ventral view, snout projects moderately beyond recessed mouth, margins of lower jaw and upper lips slightly more blunt than ST. Eyes not visible. TAs slightly elevated, their papillae visible dorsally and ventrally, distinctly closer to CMs than to nares, minimally above imaginary lines between nares and CMs. Nares small, dorsolateral, circular depressions superficially, each with deeper, more ovate aperture anteriorly, slightly closer to level of AM than to ST, almost twice as far from bottom than from top of snout and from ST in lateral view, not visible from below.

Teeth pointed, gently recurved, lacking serrations or blade-like flanges, elements of outer series much smaller posteriorly: dentaries largest, monocuspid; premaxillary-maxillary teeth large, monocuspid; vomeropalatines much smaller, more uniform in size, bicuspid, vomerine series forming a slight angle anteromedially, palatines extending posteriorly slightly further than premaxillary-maxillary series; distance between vomeropalatine and premaxillary-maxillary series anteriorly approximately the same as AM-ST in ventral view; upper series extending posteriorly distinctly beyond choanae. Palate gently arched transversely and longitudinally. Choanal apertures subcircular, separated from each other by little more than one and a half times their individual widths, lying mostly posterior to the level of the TAs. Tongue rounded, attached anteriorly, paired swellings of the tongue opposite the choanae.

Nuchal region a little more massive than adjacent body. Two collars clearly marked by three nuchal grooves, first two completely encircling body, NG1 somewhat oblique on the sides, NG2 bending slightly anteromedially on dorsum, NG3 widely incomplete ventrally; substantial TG on dorsum of C2, visible laterally. Behind collars, 108 definitive PAGs, mostly narrowly incomplete dorsally and ventrally, except for approximately first (anteriormost) and last (posteriormost) twenty complete dorsally and about first and last six complete ventrally, the last PAG slightly in front of the level of the vent. Body ends in a terminal cap posterior to approximately level with vent, a little less than two times longer than adjacent PA, unsegmented except for single transverse groove on the dorsum. First SAG present middorsally on 100th PA, none on 101st, on right only on 102nd, complete across middorsum from 103^rd^; more posterior SAGs gradually extending further ventrolaterally, none complete ventrally. AGs slightly raised in places, particularly on the inside of body curves. Vent region interrupts last two PAGs. Scales in shallow pockets in posteriormost AGs, three rows dorsally. No indications of scales in subdermal connective tissue. Body terminus slightly acuminate, narrowing only at approximately the 107th PA without obvious vertical terminal keel. In lateral view, ventral surface strongly upturned behind vent. Vent rather transverse, with five denticulations anterior and five posterior, the interdenticular creases shorter anteriorly, not in an obvious ‘disc’ and without obvious papillae.

In preservative, lilac to steel grey, with dark middorsal band (2.5–3 mm wide), abrupt transition to much paler lateral stripes and more gradual transition to slightly darker venter. Differentiation less pronounced anteriorly and posteriorly, with darker ventral colouration fading out a little anterior to the vent on the 105th PA. Dorsum of head not noticeably paler than adjacent body; ventral surface of upper jaw and periphery of lower jaw a little paler, much paler midventrally between mandibles and on C1; paler (whitish) around nares, vent, and on posterior half of terminal cap. AGs with both whitish edge and more posterior darker aglandular border, generally appear paler than the intervening skin. Numerous whitish glands visible scattered in the skin. In life (Fig. 3), colour similar, dorsal stripe a little more obvious, dorsum of head more pinkish, paler areas of upper and lower jaws almost white.

#### Variation and additional information from paratypes

The paratypes comprise seven topotypes collected by the authors in two short visits to the forest surrounding Angoulême, French Guiana in 2008 and 2010, and a specimen collected in the vicinity of St. Laurent approximately 40 km from the type locality. The latter (BMNH 2008.722) is somewhat desiccated and a little twisted and is the only specimen with some of the SAGs complete ventrally. MNHNP 2010.0190 is a little macerated with a partially everted phallus, BMNH 2008.716 was damaged during collection and has most of the upper jaws missing, and BMNH 2008.721 died in captivity, has some areas of poor preservation of the skin, and has the jaws broken to better reveal internal features of the mouth.

Variation in some meristics and morphometrics is summarised in Table 1. There are two size classes, with six mature specimens with total lengths of between 148 and 164 mm and L/W from c. 33 to c. 39, and three much smaller and less attenuate immature specimens (BMNH 2008.718–720) with total lengths from 66 to 75 mm and L/W of c. 26 to c. 29. The immature specimens are also generally much paler than the others and appear to lack annular scales. Note that total length of live specimens is always somewhat longer than lengths of the same specimens after fixation (Table 1).

The paratypes mostly agree with the holotype but there are some noteworthy differences. There is considerable variation in the convexity of the upper lip and top of the head in lateral view with none of the paratypes approaching the curvature of the holotype. The lower jaw is noticeably less robust in MNHNP 2010.0190 and BMNH 2008.722. The TAs can be just below imaginary lines between the nares and CMs (e.g. BMNH 2007.720). NG3 is less widely incomplete ventrally in BMNH 2008.722. No specimens have a definitive terminal keel but the end of the body is distinctly narrowed and somewhat nipple-shaped in dorsal or ventral views in BMNH 2008.716 and especially in BMNH 2008.717 which does have a somewhat keel-like terminus. The number of denticulations around the vent varies from 9 to 12 but deviates only slightly from the geometric pattern of the holotype with only one more denticulation anteriorly and one more or one less posteriorly. A single unpigmented papilla is present distally on each of the lateralmost anterior denticulations of BMNH 2008.716 but seemingly absent in all other specimens.

*Microcaecilia* is generally considered to lack narial plugs, the margins of which in other taxa are often at least partially demarcated by substantial grooves. In all specimens of *M. dermatophaga*, grooves are lacking, but there are substantial bulges that are correlated with the positions of the choanae and are thus in the position expected of, and might represent, narial plugs. This warrants further investigation.

Pieces of what are presumed to be remnants of termite or ant mandibles are embedded in the skin of the head of some specimens; these are particularly clear and numerous in the slightly macerated MNHNP 2010.0190 (Fig. 4), and can be seen in live specimens, for example in BMNH 2008.721 (Fig. 5). BMNH 2008.721 has many scars on the body that were not present at the time of collection so that scaring must have occurred in captivity and presumably by intraspecific biting e.g., [7]. The distance between the choanae in this specimen is about twice the width of each choana. This is larger and also somewhat easier to judge than in the holotype because of the jaws having been forced open (Fig. 4).

Colour varies with ontogeny and somewhat among adults (Fig. 5). Young specimens lack and slowly develop the pigmentation of adults. Adults may be somewhat more vividly lilac and females may change colour while caring for hatchlings (see below).

#### Distribution and ecology

In addition to the two localities where the type series was collected, two individuals of *M. dermatophaga* were found by one of us (FS) at two additional localities. First, sometime between 1995 and 1998, under a decaying log in forest on the northern edge of the settlement of Saint Jean (5° 24″ 34.5″ N, 54° 03″ 50″ W, ca. 45 m asl). This locality is approximately 10 km south by southwest of Saint Laurent du Maroni and 45 km due west of Angoulême. Second, on 20^th^ August 2012 at Cascades Voltaires (5° 01′ 52.6″ N, 54° 05′ 15.2″ W), approximately 63 km southwest of Angoulême. The animal was found in a decomposing log that contained many termites and has since been kept alive in captivity on a termite-only diet. Captive animals from Angoulême (see below) have been fed earthworms (and occasionally crickets), indicating that *M. dermatophaga* is able to take a wide range of prey.

In c. 28.5 person hours of digging during our first visit to Angoulême between 9–11^th^ May 2008, we collected nine specimens of *Microcaecilia dermatophaga*. On 24–25^th^ April 2010, 12.5 person hours of digging yielded two more specimens. The species is syntopic with the caecilians *Rhinatrema bivittatum* and *Caecilia tentaculata*. The *M. dermatophaga* were collected in two soil types [8]: orange-brown sandy loam with some grit and little organic matter (around roots of fallen trees), and chocolate brown, less sandy loam high in organic matter.

#### Reproduction and growth

We maintained three specimens collected in 2008 together in captivity. On 9th June 2010 we observed one of these on the surface at the edge of a wooden shelter with a string of five eggs (Fig. 6) that had not been present when the specimens were examined five days earlier. Further observation was not possible until 8th July 2010, at which time one adult was found together with two small hatchlings (Fig. 6) that lacked the pigmentation of adults and appeared unable to burrow. Note the difference in skin colour of the presumed mother which is paler after her young have hatched.

On 14th July 2010 the hatchlings and presumed mother were weighed and moved to an artificial nest in a fresh container of sterile soil. On the afternoon of 26^th^ July 2010 one of us (MW) observed the approximately last 30 seconds of an episode of active skin feeding, in which the normally quiescent hatchlings were moving rapidly over and around mainly the middle region of the body of their motionless mother, removing and ingesting pieces of her skin. Immediately following this the mother and young were observed for a further approximately 30 seconds during which the mother remained motionless and the young moved very slowly upon soil adjacent to the mother. This was our only direct observation of any post-hatching parental care. By 28^th^ July the mass of the presumed mother had decreased from 3.7 to 3.09 g while the combined mass of the two hatchlings increased from 0.5 to 0.89 g. After a further six days the mass of the presumed mother had fallen further to 2.85 g and the combined mass of the two young had increased to 0.93 g. At this time the young measured 73 and 75 mm in total length and were able to burrow in loose soil. This is approximately the size of the smallest free-living specimen (BMNH 2008.718) collected in the wild in May 2008, suggesting that reproduction, if seasonal, might begin as early as March or April in the wild. From this point onwards the animals were offered live foods (earthworms and crickets). Both the presumed mother and the young subsequently increased in mass such that on 17th October 2010 the presumed mother weighed 3.6 g, close to her previous mass, and the combined mass of the young was 1.79 g, and their lengths were 102 and 111 mm.

In the 20 day period when measurements were made of animals that were housed in sterile soil with no additional source of food, the combined mass of the young almost doubled, increasing by 86%. Simultaneously the mass of the presumed mother decreased by more than 20%. On 1st June 2011, almost one year after oviposition the young weighed 1.49 and 1.73 g and had a total length of 138 and 145 mm, respectively, and resembled adult specimens in colour and behaviour. This suggests that maturity might be achieved within a year in this species.

#### Etymology

To promote stability the species epithet is considered to be noun in apposition for nomenclatural purposes and is reflective of the form of parental care; from the Greek derma = skin and phago = eating.

#### Suggested english common name

Angoulême microcaecilia.

#### Nomenclatural acts

The electronic edition of this article conforms to the requirements of the amended International Code of Zoological Nomenclature, and hence the new names contained herein are available under that Code from the electronic edition of this article. This published work and the nomenclatural acts it contains have been registered in ZooBank, the online registration system for the ICZN. The ZooBank LSIDs (Life Science Identifiers) can be resolved and the associated information viewed through any standard web browser by appending the LSID to the prefix “http://zoobank.org/”. The LSID for this publication is: “urn:lsid:zoobank.org:pub: 1427A8BF-A52C-4A12-AACA-D1AE2A834121”. The electronic edition of this work was published in a journal with an ISSN, and has been archived and is available from the following digital repositories: PubMed Central, LOCKSS.

## Discussion

*Microcaecilia* was established by Taylor [9] for three species of small Neotropical caecilians, and approximately one decade later Nussbaum and Hoogmoed [10] added a fourth species. Then, following a hiatus of 30 years, four new species of *Microcaecilia* were described within the space of only a few years [6], [11], [12], [13]. Description of *M. dermatophaga* continues this recent trend of discovery and brings the current number of species of *Microcaecilia* to nine, making it, after *Caecilia*, the second most speciose genus of caecilians in South America. Remarkably, this is the first description of a new species of caecilian from French Guiana for more than 150 years since Duméril’s [14] description of *Rhinatrema unicolor*, a species that Taylor [9] transferred to *Microcaecilia*.

*Microcaecilia unicolor* has been reported as occurring in Brazil [15], Guyana [9] and Suriname [10] but the only confirmed occurrences are in French Guiana [6], [11], [13]. We are familiar with *M. unicolor* from eastern French Guiana, especially the Kaw Mountains and Nouragues. In their distribution map for the species, Lescure and Marty [16] include localities further west, including a point close to the type locality of *M. dermatophaga* in areas where we have not found *M. unicolor*, but these records are not supported with voucher specimens and we suspect that they are probably not *bona fide* records of *M. unicolor*. Based on museum records and our own fieldwork, we further suspect that the two French Guianan endemics, *M. unicolor* and *M. dermatophaga*, may be allopatric with relatively restricted ranges. We also suspect that many more species of *Microcaecilia* remain to be discovered throughout its range.

In addition to very obvious differences in annulation and adult body colour (*Microcaecilia unicolor* have very many more SAGs and are almost uniformly black), *M. dermatophaga* and *M. unicolor* differ in a suite of dental features that Wilkinson *et al.* [11] suggested characterised two species groups in the genus. Thus, whereas *M. unicolor* have monocuspid vomeropalatine teeth, a short row of premaxillarly-palatine teeth and serrated dentary teeth, *M. dermatophaga* have bicuspid vomeropalatine teeth, a long maxillopalatine tooth row and unserrated dentary teeth.

The type series of *Microcaecilia taylori* is from Suriname, north of the Amazon, and the species was originally diagnosed by Nussbaum and Hoogmoed [10] primarily on the basis of it being the only *Microcaecilia* completely lacking SAGs. We initially identified our samples from Angoulême as specifically distinct from *M. taylori* because of their possessing a few SAGs. Subsequently, Maciel and Hoogmoed [13] assigned some *Microcaecilia* (in the collections of the MPEG) from localities south of the Amazon to *M. taylori* because they found no characters to differentiate these caecilians from the type series of *M. taylori*. This necessitated a rediagnosis of *M. taylori* because some of those specimens had as many as 21 SAGs. It also necessitated reassessment of the relationship of our material to *M. taylori*.

One of us (MW) visited the MPEG in 2008 but was not granted access to the *Microcaecilia* in the collection. Thus, although we have examined the type series of *M. taylori,* we have been unable to examine any of specimens from south of the Amazon assigned to this species. Thus we have relied entirely on Maciel and Hoogmoed’s [13] account in distinguishing *M. dermatophaga* from *M. taylori sensu lato* which we do on the basis of the presence of a transverse groove on the dorsum of the first collar in the latter. This character had not been employed previously in the systematics of *Microcaecilia* but seems to be quite helpful. As the estimated species diversity of *Microcaecilia* increases, there is a growing need for the discovery of additional morphological characters and an important role for molecular data in further testing the current taxonomy of *Microcaecilia*. Given the substantial hiatus within an atypically large distribution implied by the identification of populations from South of the Amazon as *M. taylori* [13], we would like to see the hypothesis that they are conspecific with topotypic populations from Suriname tested further.

In all specimens of *M. dermatophaga* the last definitive AG is a PAG that is approximately level with the anterior of the vent. A little less than the length of the last definitive PA further behind the vent there is an additional groove dorsally. This might be considered an additional PAG delimiting a short final PA that is undivided by a SAG, (in which case the number of PAs would be one more than reported) or as a transverse groove on the terminal cap. Irrespective of the interpretation, we are struck by the constancy of the pattern of grooves at the body terminus. We expect that more attention directed at the grooves at the end of the body will yield characters that will be useful in the taxonomy of *Microcaecilia*. It may be noteworthy that although they are mostly incomplete ventrally, AGs in the vent region on *M. dermatophaga* are essentially orthoplicate, showing none of the anteroventral bending (or cramping) seen in many other caecilian species.

Kleinteich et al. [17] argued that bony closure of the upper temporal fenestra of caecilian skulls (stegokrotaphy) confers no mechanical advantage in burrowing. An alternative explanation both for stegokrotaphy and for eyes lying under bone in caecilians that are believed to be more dedicated burrowers is that this condition evolved to prevent damage to soft tissues. The presence of probable termite or ant jaw parts embedded in the skin of the head of one of the paratypes of *M. dermatophaga* (Fig. 4) and similar occasional instances in other *Microcaecilia* [6] indicates that some prey species are potentially harmful in this respect.

*Microcaecilia* is poorly known in terms of ecology, behaviour and basic natural history and nothing was reported previously regarding its reproduction. Our direct observations of captive animals demonstrate that *M. dermatophaga* is oviparous, and that the young are beneficiaries of extended parental care in which they receive nutrition via maternal dermatophagy. Our observations of complementary changes in mass of young and the attending putative mother, and of skin colour change in the latter, further support the importance of maternal dermatophagy in this species, being consistent with similar changes in the other three reported instances of this form of parental care [1], [2], [18]. Maciel and Hoogmoed [13] illustrated so-called “fetal” teeth in two small (77–78 mm total length) *Microcaecilia* (one *M. taylori* and one *M*. sp.) that they examined without commenting upon their significance. These multicuspid teeth are very similar to those used by hatchling *Siphonops annulatus* [2] to graze upon the modified skin of the attending mother. We hypothesise that the “fetal” teeth illustrated by Maciel and Hoogmoed [13] are not foetal and that they are used by the young in skin feeding. In order to minimise disturbance we made no direct observations of the teeth of young *M. dermatophaga* during their skin-feeding phase. We predict that young specimens will have multicuspid teeth similar to those reported by Maciel and Hoogmoed [13] in other *Microcaecilia*.

Maternal dermatophagy is an only relatively recently discovered behaviour in caecilians [1] reflecting both the historical lack of study of this group and the difficulty of studying reproduction in animals that spend most of their time in soil that must be disturbed for them to be observed. It is thus unsurprising that terrestrial reproduction has rarely been reported upon in caecilians [17]. Our results demonstrate the potential for basic observations in captivity to contribute substantially to our understanding of caecilian reproduction particularly when parental care continues for some extended period of time.

On the basis of their discovery of maternal dermatophagy in distantly related caecilians from Africa and South America, Wilkinson *et al.*, [2] hypothesised that this was an ancient form of parental care and predicted that it would be widespread among the many teresomatan caecilians for which information on reproduction was entirely absent or substantially incomplete. Its discovery in *Microcaecilia* is as expected from this prediction and strengthens the hypothesis that all members of the Siphonopidae are oviparous skin-feeders. We note that Kouete *et al.* [18] recently provided indirect evidence that skin feeding occurs in the African caecilian, *Herpele squalostoma*, and thus is possibly an ancestral feature also for the Herpelidae.
